# Role of RNF20 in cancer development and progression – a comprehensive review

**DOI:** 10.1042/BSR20171287

**Published:** 2018-07-13

**Authors:** Gautam Sethi, Muthu K. Shanmugam, Frank Arfuso, Alan Prem Kumar

**Affiliations:** 1 Department for Management of Science and Technology Development, Ton Duc Thang University, Ho Chi Minh City, Vietnam; 2 Faculty of Pharmacy, Ton Duc Thang University, Ho Chi Minh City, Vietnam; 3 Department of Pharmacology, Yong Loo Lin School of Medicine, National University of Singapore, 117600 Singapore; 4 Stem Cell and Cancer Biology Laboratory, School of Biomedical Sciences, Curtin Health Innovation Research Institute, Curtin University, Perth, WA 6009, Australia; 5 Cancer Science Institute of Singapore, National University of Singapore, 117599 Singapore; 6 Cancer Program, Medical Science Cluster, Yong Loo Lin School of Medicine, National University of Singapore, 119228 Singapore; 7 National University Cancer Institute, National University Health System, 119074 Singapore; 8 Curtin Medical School, Faculty of Health Sciences, Curtin University, Perth, WA 6009, Australia

**Keywords:** cancer, nuclear factor kappaB, RNF20, ubiquitins

## Abstract

Evolving strategies to counter cancer initiation and progression rely on the identification of novel therapeutic targets that exploit the aberrant genetic changes driving oncogenesis. Several chromatin associated enzymes have been shown to influence post-translational modification (PTM) in DNA, histones, and non-histone proteins. Any deregulation of this core group of enzymes often leads to cancer development. Ubiquitylation of histone H2B in mammalian cells was identified over three decades ago. An exciting really interesting new gene (RING) family of E3 ubiquitin ligases, known as RNF20 and RNF40, monoubiquitinates histone H2A at K119 or H2B at K120, is known to function in transcriptional elongation, DNA double-strand break (DSB) repair processes, maintenance of chromatin differentiation, and exerting tumor suppressor activity. RNF20 is somatically altered in breast, lung, prostate cancer, clear cell renal cell carcinoma (ccRCC), and mixed lineage leukemia, and its reduced expression is a key factor in initiating genome instability; and it also functions as one of the significant driving factors of oncogenesis. Loss of RNF20/40 and H2B monoubiquitination (H2Bub1) is found in several cancers and is linked to an aggressive phenotype, and is also an indicator of poor prognosis. In this review, we summarized the current knowledge of RNF20 in chronic inflammation-driven cancers, DNA DSBs, and apoptosis, and its impact on chromatin structure beyond the single nucleosome level.

## Introduction

Cancer is a disease that afflicts men and women, young and old, without any specific etiology. Remarkably, cancer has been studied for over more than several decades; however, the development of cancer is yet a mystery. Several risk factors have been identified as potential initiators of cancer. Development of cancer was often associated with high consumption of red meat, heavy smoking, chronic alcohol intake, viral infections, lifestyle, and environmental factors. Recent advances in research and technology have identified additional inherent risk factors that may or may not be heritable, which range from cellular allelic mutations, somatic mutations, accumulating mutations such as hot spot mutation, homozygous gene deletion, or gene amplification, non-synonymous single nucleotide polymorphisms, inflammatory tumor microenvironment, angiogenesis, and epigenetic alterations in the genome of normal cells that transforms them into cancer cells with characteristic properties such as uncontrolled cell proliferation, and are associated with invasive and metastatic potential [1–5]. These mutations are often associated with poor prognosis. The epigenetic impact in cancer development is yet a largely unexplored area and it is potentially an evolving strategy to counter the development and progression of cancer [6–12].

Several studies have indicated that cancer cells are often associated with modifications or alterations in their chromatin landscape and are associated with DNA replication and repair [13–15]. Five diverse types of DNA repair systems have been recognized based on the type of DNA damage: (i) direct reversal, (ii) base excision repair (BER), (iii) nucleotide excision repair (NER), (iv) DNA mismatch repair (MMR), and (v) double-strand break (DSB) repair [15–17]. In DNA DSB repair, the most important marker is the histone H2A variant H2AX, which is also a surrogate marker for DSB repair. Ring finger protein 20 is an E3 ligase that ubiquitinates histone H2B [18,19]. Ubiquitination of histone H2B (H2Bub1) has been demonstrated to be implicated in chromatin dynamics during transcription regulation, and previous studies have indicated that it is also involved in homologous recombination by altering chromatin structure [20]. Interestingly, it was also observed that ubiquitination of H2B promotes the accumulation of chromatin remodeling factor SNF2H in DNA repair and sustains euchromatin structure [20,21].

A RING (really interesting new gene) family of E3 ubiquitin ligases enables homo- or heterodimeric complex formations such as RNF20–RNF40, BRCA1–BARD1, BIRC7, CHIP, cIAP, IDOL, RNF4, Prp19, Mdm2–MdmX, and RING1B–Bmi [22]. In general, it is believed that the RING family of E3 ligases co-operates with E2 enzyme through the RING domain [19,22]. Both RNF20 and RNF40 contain a RING domain in their C-terminal and are orthologs of yeast BRE1 [23–25]. The RING domain is absolutely essential for ubiquitin ligase activity, formation of homo/heterodimeric complexes, and stability of E3 ubiquitin ligases [23–26]. Knockdown of either RNF20 or RNF40 leads to degradation of both the proteins [26]. In contrast, the RING domain of RNF20/40 and BRE1 is not required for interacting with RAD6 enzyme [27–29]. The present review is designed to collate the existing literature, and critically analyze our current understanding and the recent advances in RNF20/40 mediated processes and their implication in cancer development.

**Figure 1:**
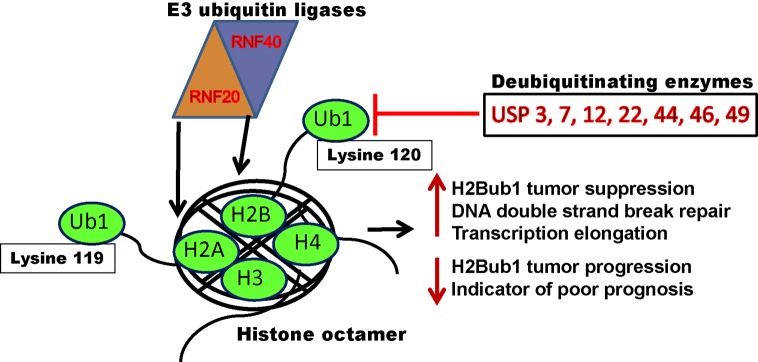
Reversible post-translational modification (PTM) ubiquitination of H2A (K119) and H2B (K120) on specific amino acid residues on core histone tails regulates various biological processes, including transcription elongation, inflammation, DNA replication, and DNA DSB repair processes, and is highly dynamic in nature These modifications are added on to histones by RNF20/40 E3 ubiquitin ligases. These PTM marks are identified and removed by active enzymes known as deubiquitinating enzymes. Any deregulation of this process often leads to malignant cellular transformation.

## RNF20 in chromatin and histones remodeling – implication in cancer phenotype gene expression

All eukaryotic cells contain identical DNA that has a unique ability to differentiate and maintain specific phenotypes and functions. The key regulators of this unique and important specific capability are the chromatin associated histones on the DNA. Several studies have demonstrated that modifications such as addition or deletion on DNA and/or histones by methylation, acetylation, SUMOylation, ADP ribosylation, ubiquitination, phosphorylation, and several other modifications on histone serine, threonine, and lysine residues or the DNA itself by specific enzymes regulating several processes such as maintaining cell identity, cell cycle regulation, proliferation, and genome integrity [6,10–12,30]. These changes in the genome of the cells are known as epigenetic changes that regulate activation or repression of gene expression [30–33]. In eukaryotic cells, 147 bps of DNA are wrapped around the core octamural globular histone proteins known as the histone octamer containing pairs of H2A, H2B, H3, and H4. The histone octamer forms the fundamental unit known as the nucleosome, which is the building block for chromatin and chromosomes [12,30,33]. Thus, the state of chromatin (i.e. euchromatin or heterochromatin formation) determines the gene expression pattern, resulting in profound changes in the cellular phenotypes and critical biological functions [9,34,35]. Deregulated epigenetic changes have been associated with the development of several diseases including chronic inflammation-driven cancers [32,33,36–39].

In cancer cells, numerous epigenetic alterations are observed in genes regulating cell cycle, oncogenes, tumor suppressor genes, and apoptosis related genes, such as aberrant methylation or acetylation of the histones and/or DNA [12,32,34,39]. Histones have been shown to be ubiquitinated and are associated with transcription regulation. Addition of ubiquitin molecules to histones can directly modulate transcription factors and their cofactors. The equilibrium between ubiquitination and deubiquitination is vital for normal cell function, and any disparity frequently leads to malignant transformation. Ubiquitin is a 76-aa polypeptide responsible for the addition of ~8.5 kDa to the overall mass of a histone (~11–15 kDa). Ubiquitin contains seven lysine (K) residues. The process of ubiquitination is an ATP-driven process involving the E1 and E2 enzymes, permitting E3 ubiquitin ligase to ubiquitinate histone directly or indirectly [12,40–42]. In general, histone ubiquitination is a natural physiological cellular process and participates in DNA DSB repair, regulation of transcription, and genome stability. Histone polyubiquitination is often not processed for proteosomal degradation; instead it regulates gene transcription [43]. Remarkably, it has been reported that histones H2A and H2B are more prone to ubiquitination [11,12,18,44,45]. Ubiquitylation of histone H2B in mammalian cells was identified in 1980 [46] (Figure 1).

H2A monoubiquitination on K119 plays a role in transcriptional silencing of polycomb proteins and in genome maintenance compared with H2B, which is ubiquitinated at K120. These ubiquitin marks can be deubiquitinated by MYSM1 (2A-DUB) [47]. In yeast, histone H2B is monoubiquitinated on lysine 123 (K123) by ubiquitin-conjugating enzyme (E2) RAD6 and ubiquitin ligase (E3) BRE1 [23–25]. H2B is ubiquitinated on its C-terminal tail and significantly increases transcript length [20,48,49]. In eukaryotic cells, RNF20/RNF40 can exclusively monoubiquitinate K120 on histone H2B [21,26]. Moreover, H2B ubiquitinations are reversible and are promptly removed by deubiquitinating enzymes (DUBs). Several DUBs have been identified that have been shown to deubiquitinate mammalian H2B, such as USP3, USP7, USP12, USP22, USP44, USP46, and USP49 [50–55]. H2B monoubiquitination (H2Bub1) plays a pivotal role in both activation of transcription and tumor suppression [56,57]. Aberrant H2Bub1 is the key to initiation of malignant transformation and directly influences chromatin structure beyond the level of the single nucleosome [58,59]. Ubiquitin-conjugating enzyme E2A (UBE 2A or RAD6A) and the RNF20/40 E3 ligase complex are responsible for catalysis in H2B ubiquitination. Deregulation in any of these process leads to the development of tumors, as evidenced with hypermethylation of RNF20 promoters in breast tumor samples [60]. Mutations in cell division cycle 73 (CDC73) lead to loss of maintenance of H2Bub1 PTM both *in vitro* and *in vivo.*

Abnormally regulated or mutated CDC73 has been reported in several tumors such as breast, colorectal, gastric, parathyroid, renal, and in patients with familial disorder-hyperparathyroidism jaw tumor syndrome [61]. Interestingly, in malignant breast cancer samples, low levels of H2Bub1 were correlated with tumor suppression compared with normal and benign samples [60]. Deregulated expression and hyperactivation of deubiquitination enzymes also upset the overall expression of H2Bub1 [50,59,62]. In addition, USP22 was found to carry homozygous gene deletions, gene amplifications, and non-synonymous single nucleotide polymorphisms in a variety of tumor types [43]. In a study by Zhang et al. [63] (2011), USP22 was found to be up-regulated in breast cancer patient samples and was associated with aggressive phenotypes and decreased levels of H2Bub1 compared with benign tumors. Furthermore, USP22 overexpression was associated with breast cancer lymph node metastasis and recurrence, and was a pointer of poor prognosis [63,64]. An *in vitro* reconstituted transcription assay demonstrated that H2B ubiquitination regulates the transcription elongation regulator PAF complex and the RNF20/40 heterodimer, indicating that RNF20 transcription regulation is complex in cells [20].

## Role of RNF20 in inflammation and inflammation-driven cancers

Chronic inflammation has been widely associated with diseases such as asthma, Alzheimer’s disease, rheumatoid arthritis, atherosclerosis, multiple sclerosis, and a variety of cancers, and is a key driver of cancer progression [4,11,65–73]. Rudolf Virchow in 1858 proposed chronic inflammation in cancer [66,69,74]. Virchow postulated that it is the tumor microenvironment that sustains persistent inflammation, which drives the initiation and development of oncogenesis [68,70,75]. Acute inflammation is mainly a self-limiting process and can be treated therapeutically; however, prolonged chronic inflammation is mostly detrimental [2,65,76,77].

Chronic inflammation is associated with the production of pro-inflammatory cytokines and chemokines that constitutively activate pro-survival transcriptional factors that act as key regulators of tumor promotion and progression [11,66,70,78]. Cancer development in the presence of chronic inflammation involves the constant presence of activated oncogenes and transcriptional factors such as nuclear factor-κB (NF-κB), signal transducer and activator of transcription 3, activator protein 1, hypoxia-inducible factor 1α, forkhead box protein M1, peroxisome proliferator associated receptor γ, Wnt/β-catenin, c-Met (hepatocyte growth factor receptor), and hedgehog (HH/GLI) [2,4,70,71,79–81]. The transcriptional factor NF-κB was discovered in 1986 by David Baltimore [82–84]. The mammalian NF-κB family of transcription factors is composed of RelA (p65), c-Rel, RelB, p50 (NF-κB1), and p52 (NF-κB2). They all share a conserved REL homology domain of ∼300 amino acids that play a pivotal role in their functions such as DNA dimerization, binding, and heterodimerization interaction with inhibitory κBs (IκBs), which are the intracellular inhibitors of NF-κB [3,4,11,71,81,85–88]. In resting cells, the majority of NF-κB complexes are primarily cytoplasmic and present in an inactive form due to their binding to the IκB family of proteins that prevent DNA binding and, as a consequence, prevent nuclear accumulation [71,77,81,85,88–90].

Ubiquitination of histone H2B is predominantly through E3 ligase RNF20 and it is found to be down-regulated in a majority of cancers [24,43,60,61,73,91–95]. Inflammation is a securely regulated process that can be very effectively turned on or off in normal physiological conditions [87,96,97]. Factors linking chronic inflammation and cancer are of great interest, and several lines of evidence suggest that the constitutive activation of pro-inflammatory transcription factors plays a pivotal role in the sustained cell proliferation observed in cancers [1,3,66,70,81]. Notably, inflammatory bowel disease patients are more prone to developing colorectal cancer [98,99]. H2Bub1 is considered to play a crucial role during transcription, and any change in levels of H2Bub1 affects the transcriptional response to epidermal growth factor [100], estrogen [60,95], interferon γ [101], and androgens [102].

A recent report by Tarcic et al. (2016) [73] showed that RNF20 depletion with a concomitant reduction in H2Bub1 augments tumor necrosis factor-induced activation of NF-κB and its subsequent pro-inflammatory cytokine and chemokine genes. They showed that mice with reduced RNF20 and H2Bub1 are more susceptible to chronic colon inflammation and colorectal cancer, which is associated with activation of NF-κBp65 and reduced H3K9 trimethylation on a subset of NF-κB target genes. *In vivo* RNF20^+/−^ mice were shown to be predisposed to acute and chronic colonic inflammation and development of colorectal cancer. Surprisingly, RNF20/40 and H2Bub1 were markedly reduced in the epithelium and stroma of ulcerative colitis patients and in human colorectal tumor samples [73]. Genetic instability has been identified to play a critical role in the development of colorectal cancer [103]. Barber et al. (2008) [103] for the first time identified five genes *SMC1L1* (two independent mutations), *CSPG6, NIPBL, STAG3*, and *RNF20* involved in sister chromatid cohesion and mutations in these genes can lead to chromosome instability in colorectal cancers.

### Breast cancer

Breast cancer is the second most common cancer that afflicts women, with an estimated 1.67 million women diagnosed with breast cancer in 2012 [5,104,105]. Breast cancer was ranked fifth in cancer-associated deaths amongst all cancers globally in 2012 [5,104]. Despite outstanding progress in the identification of specific genes involved in the breast cancer progression, our current knowledge of the complex machinations of the epigenetic landscape is still limited. The RNF20/40 heterodimeric complex is a known major E3 ligase that is responsible for H2Bub1 on K120 and also facilitates H3 methylation on K4 and K79, thereby regulating transcription [106–108]. Interestingly, in human breast cancer MCF7, MDA-MB-231, and T47D cell lines, RNF20/40 was found to be highly expressed compared with the normal human breast epithelial cell line MCF-10A [109]. In this particular study by Duan et al*.* (2015) [109] they showed that RNF20/40 is physically associated with motor protein Eg5 and is functionally involved in spindle assembly in breast cancer cells. Proteins that are involved in mitosis are often found to be overexpressed in a variety of tumor cells, primarily due to an elevated mitotic index [109–111]. Furthermore, athymic nude mice receiving MCF-7 cells infected by lentiviruses carrying empty vectors or MCF-7 cells with lentivirus-delivered Eg5, RNF20, or RNF40 knockdown, the tumor growth was significantly suppressed compared with control mice, suggesting that an RNF20/40-Eg5 axis is involved in breast carcinogenesis [109].

Protein interaction networks data show relationship between disease-causing genes is often greater than expected by chance and serves as a powerful means for the identification of new genes in diseases such as breast cancer [112–116]. *RNF20* was found to be associated with four other genes (*SNAI1, WHSC1L1, BCAS3*, and *MTA3*) that share common domains with DNA damage repair (DDR) enzymes. However, it is RNF20 that has been implicated as a modulator of DDR in breast cancer cells [116]. Monoubiquitylation of histone H2B (H2Bub1) is catalyzed primarily by the RNF20/RNF40 complex and removed by multiple DUBs.

RNF20 knockdown significantly reduces H2Bub1 expression and promotes migration in both breast cancer cells and in non-transformed mammary epithelial cells. The Let-7 family of miRNAs is a tumor suppressor that was shown to regulate H2B ubiquitination and reduced breast cancer cell migration, suggesting additional mechanisms of action may be involved in its tumor-suppressor effects [117]. Silencing of RNF20 in breast cancer cells can function as a tumor promoter [100]. It was found that depletion of RNF20 increased breast cancer cell proliferation and migration potential. It is of note that RNF20 promotes CpG island hypermethylation in several breast cancers and that down-regulation of H2B ubiquitination promotes tumorigenesis [100]. In NIH3T3 mouse cells, RNF20 silencing up-regulated formation of colonies in soft agar, indicating neoplastic transformation of cells [100]. In conclusion, up-regulation of RNF20/40-mediated up-regulation of H2Bub1 may down-regulate cancer progression and may be a therapeutic target for cancer prevention and treatment.

### Mixed-lineage leukemia-rearranged leukemia

The mixed-lineage leukemia (MLL) proto-oncogene *MLL1* was found to be involved in chromosomal translocations occurring frequently in acute myeloid leukemia (AML), acute lymphoblastic leukemia (ALL), infant acute leukemia, and in patients treated with topoisomerase II inhibitors [118]. MLL rearrangements initiate aggressive forms of acute leukemia and are associated with poor outcome [118]. In a study by Wang et al*.* (2013) [93] they reported the role of RNF20 in the pathogenesis of MLL-fusion leukemia. They found that RNF20 is an additional chromatin regulator that is necessary for MLL-fusion-mediated leukemogenesis and that suppression of RNF20 in leukemia cells leads to inhibition of cell proliferation *in vitro*. Suppression of RNF20 slowed down leukemia progression in an *in vivo* animal model and was associated with down-regulation of the *MLL-AF9* target gene [93].

In this context, another class of chromatin remodelers, histone deacetylases, has recently emerged as a promising target in MLL-rearranged ALL. Histone deacetylase inhibitors such as LBH589 (panobinostat) have shown promise as an antileukemic against MLL-rearranged infant ALL cells *in vitro*, with a promising therapeutic index and was effective at nanomolar concentrations [119]. In a recent study by Garrido et al. (2018) [120] they showed that panobinostat could inhibit tumor growth in an ALL xenograft mouse model and prolonged survival of mice. It was also reported that the antileukemic effect was targetted by the suppression of H2B ubiquitination by inhibiting the RNF20/RNF40/WAC E3 ligase complex and inducing apoptosis [120]. This finding also implicates RNF20 as a potential target including other classes of E3 ligases with existing or other new compounds that may potentially act specifically against ubiquitin ligases [121,122].

### Prostate cancer

Prostate cancer is the fourth most commonly occurring cancer globally and the second most common cancer in men [123]. Histone modification by methylation, acetylation, or ubiquitination has been reported to be deregulated in a variety of cancer cells [100]. The concomitant activation of polycomb ubiquitin ligases RNF2 and deubiquitinase USP22 is significant during cancer progression because USP22 activation allows transcriptional up-regulation of cell cycle related genes [50]. For instance, H2A ubiquitin ligase RNF2/RING1b and H2B deubiquitinase USP22 are associated with poor prognosis in numerous cancers [50]. Interestingly, genes encoding polycomb group protein BMI-1 and EZH2 are found to be amplified in metastatic prostate cancer, with a concomitant increase in levels of H2Aub1 and H3K27me3 [124]. The Oncomine database reveals that metastatic prostate cancer cells have decreased the levels of RNF20 [102]. In a previous study, it was shown that RNF20 and RNF40 interact with androgen receptor and modulate its transcritpional activity in androgen-dependent LNCaP prostate cancer cells, and depletion of RNF20 or RNF40 is strongly correlated with inhibition of LNCaP cell proliferation and a reduction in H2Bub1 levels [102].

### Lung cancer

Lung cancer is the primary cause of cancer-related deaths worldwide. Approximately 85% of lung cancers are non-small-cell lung cancer (NSCLC), while lung adenocarcinoma accounts for approximately 50% of NSCLC [125,126]. Using human lung cancer A549, H1299, and H460 cell lines, and normal lung epithelial cells, suppression of H2Bub1 by RNF20 knockdown was associated with significant decrease in H3K4 and H3K79 trimethylation. It was also observed that RNF20 knockdown and down-regulation of H2Bub1 affect several cellular signaling pathways and enhanced proliferation, migration, invasion, and cisplatin resistance of these cells [127]. Furthermore, lung cancer patients with H2Bub1-negative cancers have shorter survival outcomes compared with H2Bub1-positive patients [127]. The present study, has for the first time demonstrated that loss of H2Bub1 is associated with enhanced malignancy and poor differentiation of lung adenocarcinoma.

### Clear cell renal cell carcinoma

Clear cell renal cell carcinoma (ccRCC) is the foremost subtype of kidney cancer. Excessive lipid accumulation in the kidney is one of the characteristics of the aggressive form of ccRCC [128,129]. In general, lipid accumulation and lipogenesis is increased in several cancers [130,131], and lipid metabolites are produced by activation of lipogenesis [132,133]. Increased lipid synthesis is another hallmark of cancer [134]. Sterol regulatory element-binding protein (SREBP) plays a central role in lipid metabolism and membrane biology. Incidentally, Brown and Goldstein identified a nuclear protein that is bound to sterol regulatory element of the low-density lipoprotein receptor and controls transcription [135–137]. Subsequently, SREBP1c was demonstrated as a major determinant of adipocyte determination and differentiation [138]. SREBP targets such as fatty acid synthase and LDL-receptor are often found to be elevated in cancer cells, thus implicating SREBP in deregulated lipogenesis in cancer cells and targetting lipid supply serves as a potential target for anticancer therapy [139,140]. RNF20 has been suggested to act as tumor suppressor in chronic inflammation-driven cancer [73]. RNF20 has also been implicated in polyubiquitinating and degrading SREBP1c upon protein kinase A activation, thus down-regulating lipid metabolism [137,141].

In a recent study, RNF20 was demonstrated to have tumor suppressor activity in ccRCC. RNF20 overexpression inhibited lipogenesis and ccRCC cell proliferation by down-regulating SREBP1c [142]. Furthermore, RNF20 overexpression greatly reduced tumor growth and lipid storage in a xenograft mouse model [142]. In the clinical setting, ccRCC patients with low levels of RNF20 and SREBP1 activation have been reported to be linked with poor prognosis [142]. In addition, tumor-associated mutant p53 has been shown to bind and transcriptionally activate SREBP2 and activate the mevalonate pathway [143]; it is highly possible that p53 and SREBPs may potentially regulate each other. Furthermore, p53 has been shown to have well-known roles in oncogenesis and RNF20 has a role in DNA damage response, it would be critical to determine whether RNF20-mediated suppression of SREBP1c will impact on these pathways, and further strengthen the link between SREBPs and cancer [144].

### Other cancers

There was a loss of global H2Bub1 in 77% (313 of 407) of high-grade serous ovarian cancers and it was observed at all the stages (I–IV) of tumor development [145]. Numerous studies have implicated RNF20 in oncogenesis and it was found to be somatically mutated or deleted in various cancers including breast, colon, lung, and prostate cancer (Table 1) [7,100,146–148].

**Table 1:** RNF20 somatic alterations for a given cancer type

Cancer type	Number of new cases diagnosed in U.S.A. and Canada	Overall alterations (%) (deletions, mutations, amplification, multiple)	References
Breast	276, 989	1.1	[148,171]
Lung	252, 826	1.6	[148,183]
Prostate	202, 499	3.3	[148,184]
Colorectal	160, 640	3.3	[148,175]
Uterine	79, 607	5.8	[148,176]
Pancreatic	58, 230	3.7	[148,185]

## Role of RNF20 in DNA DSB repair

Chromatin dynamics is highly regulated by numerous intracellular signaling networks. These networks often control the extent of extracellular signal activation that helps in normal cellular homeostasis. However, deregulated signaling directly threatens genomic stability, which may result in malignant transformation or cell death [44]. Several risk factors have been identified, such as ionizing radiation, radiomimetic chemicals, environmental factors, and replication fork stalling, which potentially cause cellular genomic DNA damage and DNA DSBs [149–153]. DNA damage is often countered in normal cells by DNA damage response signaling processes. The major DSB repair pathway is the error-prone non-homologous end-joining (NHEJ) and homologous recombination between sister chromatids [154–156]. The initial response to DSB is characterized by extensive PTMs such as acetylation, phosphorylation, ubiquitination, and SUMOylation [44,157].

Dynamic alterations in chromatin and associated histone marks have been identified as important events in DSB and DNA repair mechanisms [158–160]. H2Bub1 has been demonstrated to be a critical event in DDR in eukaryotic cells [89,90,91]. H2Bub1 was first found to be induced in DSB and DDR in yeast. In yeast, the BRE1 mutant showed higher sensitivity to ionizing radiation and was associated with RAD51, a key molecule in homologous recombination repair [160]. Preliminary studies have indicated that BRE1 functions in an RAD51-dependent fashion; however, the molecular mechanisms need to be further elucidated [160]. Upon DSB, protein kinase ataxia telangiectasia mutated (ATM) catalyzes phosphorylation of HNF20 and HNF40 on serine residues. p-HNF20/40 is then recruited to the sites of DSB where it ubiquitinates DNA damage associated H2Bub1 [26]. In another study by Nakamura et al. (2011) [21] they showed that RNF20 functions with the MRE11, RAD50, and NBS1 repair complex (termed as the MRN complex) at DSB sites and augments the repair process through SNF2H-mediated chromatin reorganization [20,21]. This process of H2B ubiquitination is required for both NHEJ (XRCC4 and Ku80) and homologous recombination repair (RAD51, BRCA1, and BRCA2) mechanisms [21,26,44].

Mutated or defective NBS1 potentiates cell death upon ionizing radiation due to impaired homologous recombination and NHEJ repair mechanisms [161,162]. RNF20 was shown to interact with the several critical domains for protein–protein interaction in the C-terminal of NBS1; however, ATM interaction with RNF20/40 is yet to be reported [20,21]. Cancer cells treated with doxorubicin (a DNA damaging agent) have been linked to global loss of H2Bub1, especially those cells encoding proteins that play a pivotal role in DDR by either maintaining or increasing the levels of H2Bub1 [163]. Using a p53 overexpression model, H2Bub1 was found to be at the transcribed region of the p53 target CDKN1A and was associated with recruitment of RNA Pol II and a concomitant increase in CDKN1A [163]. Silencing of RNF20 by si/shRNA in cells augmented ionizing radiation and DNA damaging agents such as camptothecin, neocarzinostatin, and mitomycin C, with severe impairment of DNA repair mechanisms [21,26,91]. Moreover, overexpression of mutant H2B and silencing of RNF20 did not have any additional effect on cells, indicating that RNF20 functions by ubiquitinating H2B in DSB repair. In addition, RNF20 in DSB repair is correlated with euchromatin structure, strongly supporting the evidence that defects in DSB repair protein accumulation at the DSB sites were released by compounds that induce chromatin relaxation [21,164].

A study by Fierz et al. (2011) [58] showed that H2B ubiquitination obstructs chromatin compaction, resulting in an open and biochemically accessible fiber conformation. Furthermore, H2B ubiquitination by RNF20 is followed by accumulation of chromatin remodeling factor SNF2H. Indeed, several studies have shown that depletion of SNF2H sensitized cells to ionizing radiation and DNA damaging agents, and the effect was comparable with RNF20 silencing [20,21,165,166]. Recently, Klement et al. (2014) [167] reported that RNF20-SNF2H is involved in DSB repair and induces euchromatin structure in a SNF2H-dependent manner. Heterochromatin acts as a barrier to DNA repair, with a strong correlation with increased somatic mutations in cancer [167]. In cancer cells, low levels of H2Bub1 contribute to cancer progression and influence several aspects of chromatin function, including transcription regulation and DNA repair [117]. Ionizing radiation induces an overabundance of diverse types of DNA damage, of which DSB accounts for less than 5% compared with DNA single-strand breaks and DNA base damage that is more commonly observed. HeLa and oropharyngeal squamous cell carcinoma (UMSCC74A and UMSCC6) cells that were subjected to radiation demonstrated ionizing radiation-induced complex DNA damage (CDD).

It was demonstrated that H2Bub1 is specifically induced for several hours after irradiation [168]. RNF20/40 has been previously reported to be involved in DSB repair [21]. Carter et al. (2018) [168] showed that abrogation of RNF20 is strongly associated with suppression of H2Bub1 and DNA transcription. In a recent study it was shown that PARP1 inhibitors (Olaparib and BMN673) could preferentially inhibit the proliferation of RNF20-deficient cells by inducing severe DNA DSB (γ-H2AX) and apoptosis (cleaved Caspase-3) [148]. In a recent report by Guppy et al. (2017) [148] they have introduced a new approach known as synthetic lethal targetting to deliver highly specific tumor cell killing. BRCA1 and BRCA2 ubiquitin ligases are involved in DSB homologous recombination repair, also known as the error-free DNA DSB repair pathway [148,169,170]. BRCA1 and BRCA2 are mutated in breast, ovarian [171–173], prostate, lung, and colorectal cancer, and are potential biomarkers for a synthetic lethal targetting strategy [174–177].

## Conclusion

Deregulated epigenetic changes have been implicated in the development of several inflammation-driven diseases, including cancer. Numerous epigenetic alterations have been identified in histones, which determine the euchromatin or heterochromatin state that impedes critical physiological functions and may lead to malignant transformation. Ubiquitination of histones is one of the critical histone PTMs occurring on histone H2B and is regulated by the E3 ligase RNF20/40. The balance between H2Bub1 and deubiquitinating enzyme USP22 is critical and disruption leads to tumorigenesis, as H2Bub1 has been implicated in both transcription and DNA DSB repair pathways. Several lines of evidence suggest that RNF20’s role in DSB is mediated by ubiquitination of H2B. Another possibility that has been suggested is histone methylation concomitant with ubiquitination. However, there are technical difficulties in achieving this as it has been shown that the human genome has at least 17 RNF20 genes and would require recently developed techniques such as CRISPR-Cas9 and TALEN to overcome these problems [178–182]. RNF20 has been suggested to act as a tumor suppressor in chronic inflammation-driven cancer.

Interestingly, RNF20 depletion has been shown to enhance NF-κB-dependent gene transcription, and TNF-mediated H2Bub1 down-regulation augments NF-κB’s response in the up-regulation of proinflammatory cytokines or chemokines that may act in an autocrine or paracrine fashion to sustain the prosurvival gene expression in cancer cells. RNF20 now adds another element by which histone polyubiquitination is often not processed for proteosomal degradation; instead it regulates gene transcription, thereby impacts the lifespan of NF-κB-p65 regulated proinflammatory genes and may regulate SREBP1c degradation. The exact molecular role of RNF20 varies in different types of cancers; therefore it is valuable to determine the role for DUBs, protein kinases, and/or pharmacological drugs in modulating RNF20 levels that may provide clinical benefits. Targetting these pathways may ultimately be exploited for cancer treatment. However, several additional *in vitro, in vivo*, and transgenic rodent studies will determine if RNF20/40 and H2Bub1 can be used as a promising target to be exploited for the prevention and treatment of cancer. Therefore, addressing these questions will hopefully advance our understanding on the role of RNF20/40 and H2Bub1 in chromatin remodeling during transcription and DNA DSB repair, and the potential of epigenetics based therapies for cancer.

## Abbreviations

ALL: acute lymphoblastic leukemia
ATM: ataxia telangiectasia mutated
BARD: BRCA1 associated RING domain
BIRC: baculoviral IAP repeat containing
BMI: BMI proto-oncogene, polycomb ring finger
BRCA: breast cancer gene
ccRCC: clear cell renal cell carcinoma
CDC73: cell division cycle 73
CHIP: carboxyl terminus of HSC70-interacting protein
cIAP: cellular inhibitor of apoptosis
CRISPR-Cas9: clustered regularly interspaced short palindromic repeats, associated protein-9 nuclease
DSB: double-strand break
DUB: deubiquitinating enzyme
H2Bub1: ubiquitination of histone H2B
IκB: inhibitory κB
IDOL (MYLIP): myosin regulatory light chain interacting protein
LDL: low-denisty lipoprotein
MLL: mixed-lineage leukemia
NF-κB: nuclear factor-κB
NHEJ: non-homologous end-joining
NSCLC: non-small-cell lung cancer
POL II: RNA polymerase II
PRP19: E3 ubiquitin protein ligase PRP19
PTM: post-translational modification
RING: really interesting new gene
RFN20: E3 ubiquitin ligases
SREBP: sterol regulatory element-binding protein
TALEN: transcription activator-like effector nuclease
